# Levels of 91 circulating inflammatory proteins and risk of lumbar spine and pelvic fractures and peripheral ligament injuries: a two-sample mendelian randomization study

**DOI:** 10.1186/s13018-024-04637-8

**Published:** 2024-03-01

**Authors:** Huiyu Huang, Zhaojun Fu, Min Yang, Haigang Hu, Chao Wu, Lun Tan

**Affiliations:** 1 Emergency Department, Zigong Fourth People’s Hospital, Zigong, China; 2 Neurology Department, Zigong First People’s Hospital, Zigong, China; 3 Orthopaedic Center, Zigong Fourth People’s Hospital, Zigong, China; 4 Digital Medical Center, Zigong Fourth People’s Hospital, Zigong, China

**Keywords:** Mendelian randomization, Fracture risk, Circulating inflammatory protein, Ligament injury

## Abstract

**Objective:**

Lumbar spine and pelvic fractures(LPF) are combined with peripheral ligament injuries(PLI), frequently. It has been reported that the site of fracture injury is usually paralleled by the secretion of inflammatory proteins. This study aimed to investigate the causal relationship between 91 circulating inflammatory proteins and LPF and PLI by using a Two-sample Mendelian randomization (MR) analysis.

**Methods:**

Single nucleotide polymorphisms (SNPs) associated with 91 circulating inflammatory proteins, as exposures were selected from a large genome-wide association study (GWAS). The genetic variant data for LPF and PLI as outcomes from the FinnGen consortium. The inverse-variance-weighted (IVW) method was utilized as the main analysis for exposures and outcomes. In addition, the final results were reinforced by the methods of MR Egger, weighted median, simple mode, and weighted mode. The sensitivity analyses were used to validate the robustness of results and ensure the absence of heterogeneity and horizontal pleiotropy. MR-Steiger was used to assess whether the causal direction was correct to avoid reverse causality.

**Results:**

This study has shown that Beta-nerve growth factor(Beta-NGF) and Interferon gamma(IFN-gamma) are both involved in the occurrence of LPF and PLI, and they are reducing the risk of occurrence(OR:0.800, 95%CI: 0.650–0.983; OR:0.723, 95%CI:0.568–0.920 and OR:0.812, 95%CI:0.703–0.937; OR:0.828, 95%CI:0.700–0.980). Similarly, Axin-1 and Sulfotransferase 1A1 (SULT-1A1) were causally associated with LPF(OR:0.687, 95%CI:0.501–0.942 and OR:1.178,95%CI:1.010–1.373). Furthermore, Interleukin-4(IL-4), Macrophage inflammatory protein 1a(MIP-1a), and STAM binding protein(STAM-BP) were causally associated with PLI(OR:1.236, 95% CI: 1.058–1.443; OR:1.107, 95% CI: 1.008–1.214 and OR:0.759, 95% CI: 0.617–0.933). The influence of heterogeneity and horizontal pleiotropy were further excluded by sensitivity analysis.

**Conclusion:**

This study provides new insights into the relationship between circulating inflammatory proteins and LPF and PLI, and may provide new clues for predicting this risk.

**Supplementary Information:**

The online version contains supplementary material available at 10.1186/s13018-024-04637-8.

## Introduction

Lumbar spine and pelvic fractures(LPF) are frequently observed in high-energy injuries, such as falls from heights, traffic accidents, and military combat [1, 2]. However, with the change in modern lifestyle, low-energy injury is increasingly common. In addition to the common loss of bone mineral density and pathological fracture, the mechanism also includes changes in the axial load of the spine [3–5]. In the anatomy of the lumbar spine and pelvis, the iliolumbar ligament begins at the inferior border of the transverse process of the 4th lumbar vertebra and the tip of the transverse process of the 5th lumbar vertebra and ends at the inner lip of the iliac crest [6]. Normally, the iliolumbar ligaments are in a role to share the load and reduce the pressure on the lumbar spine [7]. However, injury to the iliolumbar ligaments could lead to altered load distribution in the lumbar spine, excessive stress, disc degeneration, pelvic tilt, and increased risk of fracture of associated structures [8–10]. It is a potential source of increased socio-economic burden.

Inflammation is the host’s physiological response to infection or injury. However, aberrant inflammatory responses lead to tissue damage. They are central to the pathogenesis of a variety of diseases, including sepsis, autoimmunity, and atherothrombosis [11]. At present, there are more studies have shown that inflammation proteins are one of the promoted healing of fracture initiation factors, such as bone morphogenetic proteins, platelet-derived growth factor, and transforming growth factor [12, 13]. Although current studies have demonstrated that inflammatory proteins are involved in fracture healing. Whether inflammatory proteins are related to fracture risk is also a question that we need to think about, even in terms of genetic inheritance.

Mendelian randomization (MR) is a method of causal inference based on genetic variation. The basic principle is to use the effect of randomly assigned genotypes on phenotype to infer the effect of biological factors on disease [14]. This approach reduces the influence of confounding factors, unrelated to lifestyle, disease process, or environmental factors [15]. In recent years, MR has been widely used to verify causality between different exposures and outcomes [16, 17]. Therefore, data mining was performed in the latest genome-wide association study(GWAS) database and investigated the causal relationship between 91 circulating inflammatory proteins and LPF and PLI by using a Two-sample MR analysis. Meanwhile, the role of iliolumbar ligaments in lumbar spine and pelvic biomechanics is explored.

## Methods

### Study design

**Fig. 1:**
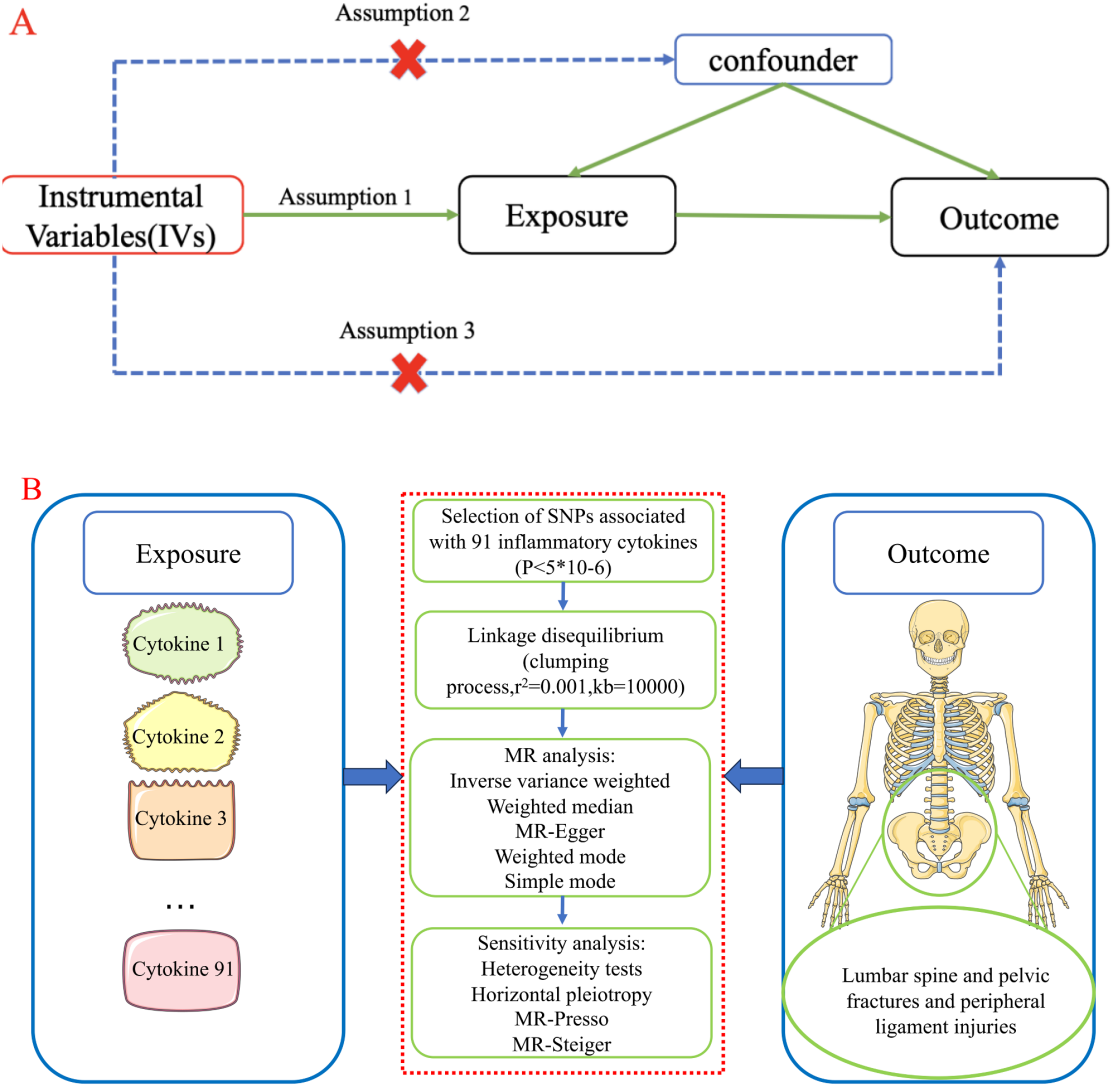
**A**: Assumption 1(relevance assumption), Assumption 2(independence assumption), and Assumption 3(exclusion restriction assumption); **B**: The study design of two-sample MR analysis

This study aimed to investigate the causal relationship between 91 circulating inflammatory proteins and LPF and PLI by using a Two-sample MR analysis. In MR analysis, three core assumptions must be met in order to obtain valid results, as shown in Fig. 1. Specifically, to be used as instrumental variables(IVs) for a risk factor, a genetic variant must satisfy (1) a reliable association with the risk factor under study (relevance assumption); (2) no association with any known or unknown confounders (independence assumption); (3) affecting the outcome only through risk factors and not through any other direct causal pathway (exclusion restriction assumption) [18].

### Data sources

The datasets used in this study were all from publicly available GWAS data summaries.

About the LPF and PLI of the database from FinnGen consortium (R9) [19], including 8812 cases and 425,678 controls from European descent. The 91 circulating inflammatory proteins were from a meta-analysis of 11 cohorts with a total of 14,824 participants of European ancestry, and the original publication provides a detailed description of the methods used to measure inflammatory proteins [11]. Full per-protein GWAS summary statistics are available for download at https://www.phpc.cam.ac.uk/ceu/proteins and the EBI GWAS Catalog (accession numbers GCST90274758 to GCST90274848). Population selection between exposure and outcome groups will not overlap. All original studies obtained ethical approval and informed consent. Details of the included GWASs are summarized in Supplementary Table S1.

### Genetic instrumental variables selection

Based on the three core assumptions of MR analysis, it is critical to ensure that the single nucleotide polymorphisms (SNPs) selected as IVs are strongly correlated with exposures. Thus, we did the following steps. Firstly, the SNPs of outcomes and 91 circulating inflammatory proteins were identified by the significance threshold of *p* < 5 × 10 − 8. For some inflammatory proteins, however, the determination of the number of SNPs is limited under that condition. To obtain more positive SNPs, we lowered the threshold (5 × 10 − 6) [20]. Secondly, the SNPs were clumped to remove linkage disequilibrium(kb = 10,000, r^2^ = 0.001) [21]. In the harmonizing process, SNPs were excluded if they were non-concordant or palindromic with intermediate allele frequencies. Finally, we calculated the strength of each SNP by the F-statistic, and SNPs with an F-statistic > 10 were considered strongly correlated [22].

### Mendelian randomization and sensitive analysis

**Fig. 2:**
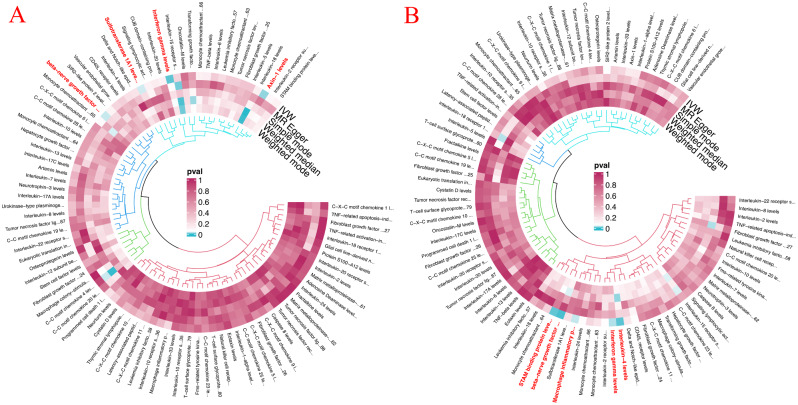
**A**: Circle diagram of 91 circulating inflammatory proteins on LPF; **B**: Circle diagram of 91 circulating inflammatory proteins on PLI. LPF: Lumbar spine and pelvic fractures; PLI: peripheral ligament injuries

The method of Inverse variance-weighted(IVW) showed the highest statistical efficacy and validity, provided that there was no pleiotropy in the IVs [23]. Therefore, IVW was used as the main research method in this study [24]. In addition, the final results were reinforced by the methods of MR Egger, weighted median, simple mode, and weighted mode [25, 26] (Fig. 2). Meanwhile, to meet the robustness of the results, Cochran’s Q test was used to evaluate the heterogeneity of SNPs in IVW and MR Egger [27]. The horizontal pleiotropy was assessed by MR-Egger intercept [25], and Leave-one-out analyses were performed to assess whether causal effects were driven by a single potentially influential SNP [28]. MR-Presso was used to detect pleiotropic residuals and outliers. MR-Steiger was used to assess whether the causal direction was correct, TURE if the exposure was likely to have caused the outcome, or FALSE if the exposure was unlikely to have caused the outcome [29]. All statistical analyses were performed in TwoSampleMR in R software.

## Results

### Influence of 91 circulating inflammatory proteins on LPF

**Fig. 3:**
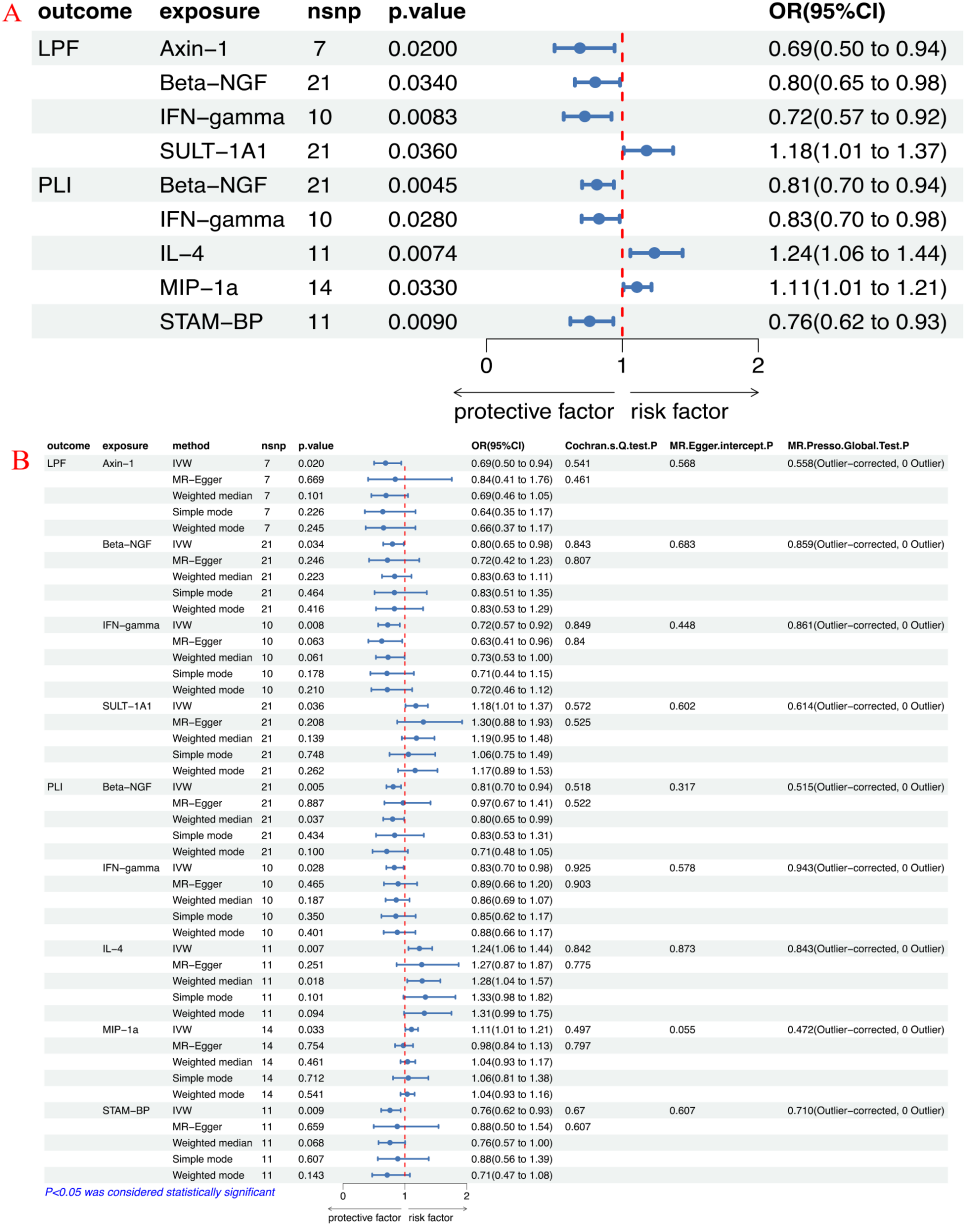
**A**: Forest plots of circulating inflammatory proteins on LPF and PLI; **B**: Forest plot of MR Results for causal association of circulating inflammatory proteins with LPF and PLI. LPF: Lumbar spine and pelvic fractures; PLI: peripheral ligament injuries

According to the IVW results, elevated levels of Axin-1, Beta-NGF, and IFN-gamma were found to be associated with a reduced risk of LPF(OR:0.687, 95%CI:0.501–0.942, *P* = 0.020; OR:0.800, 95%CI: 0.650–0.983, *P* = 0.034 and OR:0.723, 95%CI:0.568–0.920, *P* = 0.008). Conversely, heightened levels of SULT-1A1 may be linked to an increased risk of LPF(OR:1.178,95%CI:1.010–1.373, *P* = 0.036). As shown in Table 1; Fig. 3A-B.

### Influence of 91 circulating inflammatory proteins on PLI

According to the IVW results, elevated levels of Beta-NGF, IFN-gamma and STAM-BP were found to be associated with a reduced risk of PLI(OR:0.812, 95%CI:0.703–0.937, *P* = 0.005; OR:0.828, 95%CI:0.700–0.980, *P* = 0.028 and OR:0.759, 95% CI: 0.617–0.933, *P* = 0.009). Conversely, heightened levels of IL-4 and MIP-1a may be linked to an increased risk of PLI(OR:1.236, 95% CI: 1.058–1.443, *P* = 0.007 and OR:1.107, 95% CI: 1.008–1.214, *P* = 0.033). These findings are presented in Table 1; Fig. 3A-B.

### Sensitive analysis

**Fig. 4:**
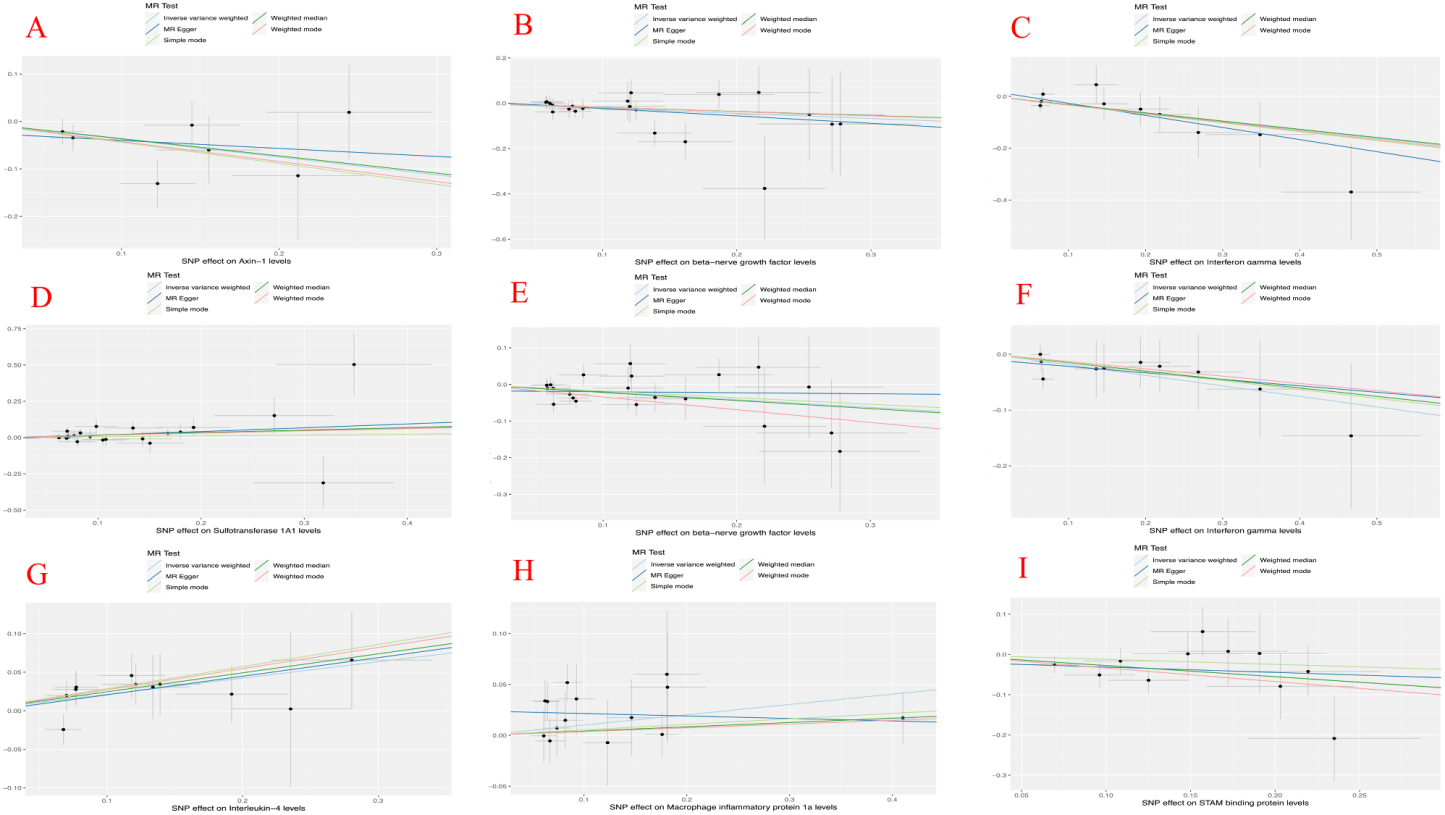
**A**-**D**: Axin-1, BNGF, IFN-gamma and SULT-1A1 of lumbar-pelvic fractures with scatter plots respectively; **E**-**I**: BNGF, IFN-gamma, IL-4, MIP-1a and STAM-BP of ligament injuries with scatter plots test respectively

Moreover, as shown in Table 2; Fig. 3A-B, no heterogeneity of SNPs was observed in IVW and MR-Egger analyses based on Cochran’s Q test. MR-Egger intercept showed no evidence of horizontal pleiotropy in this study. No outliers were detected using the MR-Presso methodology. Furthermore, there were no SNPs with a large effect size biased the estimation through the Leave-one-out test(Supplemental Fig. 1). The forest plots illustrated the causal effects of individual SNP for 91 circulating inflammatory proteins on LPF and PLI risk (Supplemental Fig. 2). Additionally, scatter and funnel plots ruled out the possibility of potential outliers and horizontal pleiotropy (Fig. 4 and Supplemental Fig. 3). The results from the MR-Steiger analysis confirmed the directionality as true without any evidence of reverse causality. The influence of heterogeneity and horizontal pleiotropy were further excluded by sensitivity analysis and the results are reliable.

**Table 1:** MR analysis of the causal association between circulating inflammatory proteins and risk of LPF and PLI

inflammatory proteins	Outcomes	SNPs	IVW				MR-Egger				Weighted median				Simple mode				Weighted mode		
			SE	OR(95%CI)	p value		SE	OR(95%CI)	p value		SE	OR(95%CI)	p value		SE	OR(95%CI)	p value		SE	OR(95%CI)	p value
Axin-1	LPF	7	0.161	0.69(0.50 to 0.94)	0.020		0.373	0.84(0.41 to 1.76)	0.669		0.221	0.69(0.46 to 1.05)	0.101		0.327	0.64(0.35 to 1.17)	0.226		0.328	0.66(0.37 to 1.17)	0.245
Beta-NGF		21	0.105	0.80(0.65 to 0.98)	0.034		0.275	0.72(0.42 to 1.23)	0.246		0.148	0.83(0.63 to 1.11)	0.223		0.247	0.83(0.51 to 1.35)	0.464		0.222	0.83(0.53 to 1.29)	0.416
IFN-gamma		10	0.123	0.72(0.57 to 0.92)	0.008		0.217	0.63(0.41 to 0.96)	0.063		0.170	0.73(0.53 to 1.00)	0.061		0.234	0.71(0.44 to 1.15)	0.178		0.248	0.72(0.46 to 1.12)	0.210
SULT-1A1		21	0.078	1.18(1.01 to 1.37)	0.036		0.201	1.30(0.88 to 1.93)	0.208		0.116	1.19(0.95 to 1.48)	0.139		0.176	1.06(0.75 to 1.49)	0.748		0.133	1.17(0.89 to 1.53)	0.262
Beta-NGF	PLI	21	0.073	0.81(0.70 to 0.94)	0.005		0.191	0.97(0.67 to 1.41)	0.887		0.105	0.80(0.65 to 0.99)	0.037		0.226	0.83(0.53 to 1.31)	0.434		0.201	0.71(0.48 to 1.05)	0.100
IFN-gamma		10	0.086	0.83(0.70 to 0.98)	0.028		0.151	0.89(0.66 to 1.20)	0.465		0.114	0.86(0.69 to 1.07)	0.187		0.160	0.85(0.62 to 1.17)	0.350		0.148	0.88(0.66 to 1.17)	0.401
IL-4		11	0.079	1.24(1.06 to 1.44)	0.007		0.197	1.27(0.87 to 1.87)	0.251		0.104	1.28(1.04 to 1.57)	0.018		0.159	1.33(0.98 to 1.82)	0.101		0.148	1.31(0.99 to 1.75)	0.094
MIP-1a		14	0.047	1.11(1.01 to 1.21)	0.033		0.076	0.98(0.84 to 1.13)	0.754		0.057	1.04(0.93 to 1.17)	0.461		0.143	1.06(0.81 to 1.38)	0.712		0.059	1.04(0.93 to 1.16)	0.541
STAM-BP		11	0.106	0.76(0.62 to 0.93)	0.009		0.290	0.88(0.50 to 1.54)	0.659		0.152	0.76(0.57 to 1.00)	0.068		0.233	0.88(0.56 to 1.39)	0.607		0.212	0.71(0.47 to 1.08)	0.143

**Table 2:** Sensitive analysis of the causal association between circulating inflammatory proteins and risk of LPF and PLI

Inflammatory proteins	Outcomes	SNPs	Cochran’s Q test			MR-Egger intercept			MR-Presso			MR-Steiger
			IVW	MR Egger		Egger intercept	p value		Global Test RSSobs	p value		causal direction
Axin-1	LPF	7	0.541	0.461		-0.023	0.568		7.120	0.558(Outlier-corrected, 0 Outlier)		TRUE
Beta-NGF		21	0.843	0.807		0.010	0.683		15.440	0.859(Outlier-corrected, 0 Outlier)		TRUE
IFN-gamma		10	0.849	0.840		0.020	0.448		5.720	0.861(Outlier-corrected, 0 Outlier)		TRUE
SULT-1A1		21	0.572	0.525		-0.011	0.602		19.334	0.614(Outlier-corrected, 0 Outlier)		TRUE
Beta-NGF	PLI	21	0.518	0.522		-0.017	0.317		22.318	0.515(Outlier-corrected, 0 Outlier)		TRUE
IFN-gamma		10	0.925	0.903		-0.010	0.578		4.631	0.943(Outlier-corrected, 0 Outlier)		TRUE
IL-4		11	0.842	0.775		-0.003	0.873		6.709	0.843(Outlier-corrected, 0 Outlier)		TRUE
MIP-1a		14	0.497	0.797		0.024	0.055		17.641	0.472(Outlier-corrected, 0 Outlier)		TRUE
STAM-BP		11	0.670	0.607		-0.018	0.607		8.995	0.710(Outlier-corrected, 0 Outlier)		TRUE

## Discussion

At present, studies have shown that there is a relationship between LPF and PLI with inflammatory proteins [12, 30, 31]. However, due to the limitations of the study, the exact causal relationship is still uncertain at the genetic level. In this exploratory study, a two-sample MR analysis was used to comprehensively assess the potential causal relationship of 91 circulating inflammatory proteins with LPF and PLI. It aims to provide more reliable evidence for clinical decision-making.

This study has shown that Beta-NGF and IFN-gamma are both involved in the occurrence of LPF and PLI, and there was a negative association. Similarly, the level of Axin-1 was negatively correlated with the risk of LPF. The level of SULT-1A1 is positively correlated with the risk of LPI. Furthermore, high levels of IL-4 and MIP-1a are positively associated with the risk of PLI and there was a negative correlation between STAM-BP levels.

The inflammatory hypothesis of aging proposes that aging is an accumulation of damage, in part due to chronic activation of inflammatory processes. The results showed that subjects with the highest number of inflammatory markers had the highest risk of fracture [32]. Similarly, Cauley et al. measured interleukin-6(IL-6), C-reactive protein (CRP), tumor necrosis factor-alpha (TNFα), soluble receptors of IL-6, TNF (TNFαSR1 and TNFαSR2), and interleukin-10(IL-10) levels in humans suggest that inflammation may play an important role in the etiology of fractures in elderly men [33]. In addition, Panuccio et al. suggested that TNF-α was significantly associated with the incidence of fractures [34]. Meanwhile, a recent study reported an association between IL-6 and hip fracture [30, 31]. Similarly, IL-10, interleukin-8(IL-8), IL-6, interleukin-1RA (IL-1RA), and monocyte chemoattractant protein-1 (MCP-1) have been shown to be associated with fracture [35]. Although these studies have elucidated that inflammatory proteins may be involved in fractures, exact causality remains challenging due to confounding variables, which may lead to bias.

Osteoblast differentiation is positively regulated by classical Wnt signaling at different stages, but high levels of β-catenin inhibit osteoclast differentiation, and Axin-1 is the main coordinator of the β-catenin destruction complex. Paulien et al. found that homozygous truncating variants in Axin-1 cause sclerosing bone disease of hip dysplasia due to loss of its C-terminal DIX domain [36]. IFN-gamma is a cytokine produced by immune cells and mesenchymal stem cells in the bone microenvironment [37]. In animal model experiments, bone histomorphometry in mice with low levels of IFN-gamma showed a pattern of low bone turnover, reduced bone formation, significantly reduced osteoblast and osteoclast numbers, and decreased circulating levels of bone formation and resorption markers [38]. The beta-nerve growth factor can stimulate cell division, growth, and differentiation. In articular cartilage, they regulate the development and homeostasis of articular cartilage by regulating the local microenvironment [39]. Additionally, the formation and healing of bone tissue are considered to be related to the development and maintenance of the nervous system. Mature bone tissue is dominated by abundant nerve fibers. Lack of nerve fiber innervation, bone growth retardation, and pain reduction. Beta-nerve growth factor induces the development of nerve fibers into bone tissue [40]. In this study, high levels of Axin-1, Beta-NGF, and IFN-gamma were observed to be associated with a decreased risk of LPF. This is consistent with the results of our study. SULT-1A1, a member of the sulfotransferase family, is located in the cytoplasm of cells and has the characteristics of a superfamily. It is significantly upregulated in inflammation, fibrosis, and cancer [41, 42]. SULT-1A1 was causally associated with LPF in this study, which may provide a new perspective on the relationship between fractures and inflammation proteins. However, further studies are needed to fully understand the specific mechanisms of inflammatory protein and fracture risk described above and to provide more evidence for potential therapeutic strategies.

Bone tissue forms a stable whole with surrounding muscles and ligaments. Ligament injury is accompanied by biomechanical changes, which will increase the risk of fracture. In this study, the functional and biomechanical mechanisms of the iliolumbar ligaments in the lumbar-pelvic region are closely related to stability, support, postural control, and motor control, which are important for maintaining the normal structure and function of the lumbar spine [43]. Likewise, this applies to other ligaments in the lumbar spine and pelvic region. Lower lumbar burst fractures (L3-L5) account for a small proportion of all spinal fractures. The iliolumbar ligament and the position below the pelvic rim are the two stabilizing factors in this type of fracture and are unique compared to burst fractures at the thoracolumbar junction [44]. The bony integrity of the pelvis is supported by a variety of ligaments, such as the posterior sacroiliac, anterior, iliolumbar, sacrospinous, and sacrotuberous ligaments, which play a crucial role in pelvic stabilization [45, 46]. Therefore, this study also explored the causal relationship between inflammatory proteins and PLI by two-sample MR analysis. Studies have shown that Beta-NGF and IFN-gamma are both involved in the occurrence of LPF and PLI. This also indirectly proves the causal relationship between inflammatory proteins and fractures, while providing clinical support for the theory of lumbar spine and pelvic systems.

## Conclusion

In this study, we employed MR Analysis to provide new insights into the relationship between circulating inflammatory proteins and LPF and PLI. That may provide new clues for predicting this risk. However, further studies are needed to fully understand the exact biological mechanisms involved.

### Limitation

The bias introduced by confounding and reverse causality was addressed by MR Analysis in this study. MR Analysis, compared with traditional observational studies, provides stronger evidence for evaluating the causal relationship between 91 circulating inflammatory proteins and LPF and PLI. Meanwhile, it provides a new research perspective. However, it is important to acknowledge that this study has certain limitations. Firstly, only European ancestry was included in the study, and further investigation is needed to determine the generalizability of the results to other populations. Secondly, the sample size of the GWAS database in this study was limited, which may have limited the statistical power of the MR analysis. Finally, While we used powerful tools to estimate the association between exposure and outcome, what has to be acknowledged is the slight sample overlap between exposures and outcomes.

### Electronic supplementary material

Below is the link to the electronic supplementary material.

Supplementary Material 1: 91 circulating inflammatory protein number names(Supplementary Table S2) and further details are provided in the Supplementary Information

## Data Availability

No datasets were generated or analysed during the current study.

## Abbreviations

MR: Mendelian randomization
SNPs: Single nucleotide polymorphisms
GWAS: genome-wide association study
IVW: inverse-variance-weighted
Beta-NGF: Beta-nerve growth factor
IFN-gamma: Interferon gamma
SULT-1A1: Sulfotransferase 1A1
IL-4: Interleukin-4
MIP-1a: Macrophage inflammatory protein 1a
STAM-BP: STAM binding protein
IVs: instrumental variables
IL-6: interleukin-6
CRP: C-reactive protein
TNFα: tumor necrosis factor-alpha
IL-10: interleukin-10
IL-8: interleukin-8
IL-1RA: interleukin-1RA
MCP-1: monocyte chemoattractant protein-1
TNF: Tumour necrosis factor
LPF: Lumbar spine and pelvic fractures
PLI: peripheral ligament injuries

## References

[1] Newell N, Pearce AP, Spurrier E (2018). Analysis of isolated transverse process fractures sustained during blast-related events [J]. J Trauma Acute Care Surg.

[2] Lei J, Zhu F, Jiang B, Wang Z (2018). Underbody blast effect on the pelvis and lumbar spine: a computational study [J]. J Mech Behav Biomed Mater.

[3] Asahi R, Nakamura Y, Kanai M (2022). Association with sagittal alignment and osteoporosis-related fractures in outpatient women with osteoporosis [J]. Osteoporos Int.

[4] Yoganandan N, Moore J, Humm J (2022). Loading rate effect on tradeoff of fractures from pelvis to lumbar spine under axial impact loading [J]. Traffic Inj Prev.

[5] Kaufman RP, Ching RP, Willis MM (2013). Burst fractures of the lumbar spine in frontal crashes [J]. Accid Anal Prev.

[6] Basadonna PT, Gasparini D, Rucco V (1996). Iliolumbar ligament insertions. In vivo anatomic study [J]. Spine (Phila Pa 1976).

[7] Yamamoto I, Panjabi MM, Oxland TR, Crisco JJ (1990). The role of the iliolumbar ligament in the lumbosacral junction [J]. Spine (Phila Pa 1976).

[8] Luk KD, Ho HC, Leong JC (1986). The iliolumbar ligament. A study of its anatomy, development and clinical significance [J]. J Bone Joint Surg Br.

[9] Aihara T, Takahashi K, Ono Y, Moriya H (2002). Does the morphology of the iliolumbar ligament affect lumbosacral disc degeneration? [J]. Spine (Phila Pa 1976).

[10] Pool-Goudzwaard A, Van Hoek G, Mulder P (2003). The iliolumbar ligament: its influence on stability of the sacroiliac joint [J]. Clin Biomech (Bristol Avon).

[11] Zhao JH, Stacey D, Eriksson N (2023). Genetics of circulating inflammatory proteins identifies drivers of immune-mediated disease risk and therapeutic targets [J]. Nat Immunol.

[12] Walters G, Pountos I, Giannoudis PV (2018). The cytokines and micro-environment of fracture haematoma: current evidence [J]. J Tissue Eng Regen Med.

[13] Pape HC, Marcucio R, Humphrey C (2010). Trauma-induced inflammation and fracture healing [J]. J Orthop Trauma.

[14] Evans DM, Davey Smith G (2015). Mendelian randomization: New Applications in the coming age of hypothesis-free causality [J]. Annu Rev Genomics Hum Genet.

[15] Burgess S, Dudbridge F, Thompson SG (2016). Combining information on multiple instrumental variables in mendelian randomization: comparison of allele score and summarized data methods [J]. Stat Med.

[16] Larsson SC, Burgess S (2022). Appraising the causal role of smoking in multiple diseases: a systematic review and meta-analysis of mendelian randomization studies [J]. EBioMedicine.

[17] Nethander M, Coward E, Reimann E (2022). Assessment of the genetic and clinical determinants of hip fracture risk: genome-wide association and mendelian randomization study [J]. Cell Rep Med.

[18] Emdin CA, Khera AV, Kathiresan S (2017). Mendelian Randomization [J] JAMA.

[19] Kurki MI, Karjalainen J, Palta P (2023). FinnGen provides genetic insights from a well-phenotyped isolated population [J]. Nature.

[20] Burgess S, Butterworth A, Thompson SG (2013). Mendelian randomization analysis with multiple genetic variants using summarized data [J]. Genet Epidemiol.

[21] Genomes Project C, Auton A, Brooks LD (2015). A global reference for human genetic variation [J]. Nature.

[22] Burgess S, Thompson SG, Collaboration CCG (2011). Avoiding bias from weak instruments in mendelian randomization studies [J]. Int J Epidemiol.

[23] Burgess S, Davey Smith G, Davies NM (2019). Guidelines for performing mendelian randomization investigations: update for summer 2023 [J]. Wellcome Open Res.

[24] Slob EAW, Burgess S (2020). A comparison of robust mendelian randomization methods using summary data [J]. Genet Epidemiol.

[25] Bowden J, Davey Smith G, Burgess S (2015). Mendelian randomization with invalid instruments: effect estimation and bias detection through Egger regression [J]. Int J Epidemiol.

[26] Bowden J, Davey Smith G, Haycock PC, Burgess S (2016). Consistent estimation in mendelian randomization with some Invalid instruments using a weighted median estimator [J]. Genet Epidemiol.

[27] Greco MF, Minelli C, Sheehan NA, Thompson JR (2015). Detecting pleiotropy in mendelian randomisation studies with summary data and a continuous outcome [J]. Stat Med.

[28] Burgess S, Bowden J, Fall T (2017). Sensitivity analyses for robust causal inference from mendelian randomization analyses with multiple genetic variants [J]. Epidemiology.

[29] Hemani G, Tilling K, Davey Smith G (2017). Orienting the causal relationship between imprecisely measured traits using GWAS summary data [J]. PLoS Genet.

[30] Saribal D, Hocaoglu-Emre FS, Erdogan S (2019). Inflammatory cytokines IL-6 and TNF-alpha in patients with hip fracture [J]. Osteoporos Int.

[31] Barbour KE, Lui LY, Ensrud KE (2014). Inflammatory markers and risk of hip fracture in older white women: the study of osteoporotic fractures [J]. J Bone Min Res.

[32] Cauley JA, Danielson ME, Boudreau RM (2007). Inflammatory markers and incident fracture risk in older men and women: the Health aging and body composition study [J]. J Bone Min Res.

[33] Cauley JA, Barbour KE, Harrison SL (2016). Inflammatory markers and the risk of hip and vertebral fractures in men: the osteoporotic fractures in men (MrOS) [J]. J Bone Min Res.

[34] Panuccio V, Enia G, Tripepi R (2012). Pro-inflammatory cytokines and bone fractures in CKD patients. An exploratory single centre study [J]. BMC Nephrol.

[35] Haller JM, Mcfadden M, Kubiak EN, Higgins TF (2015). Inflammatory cytokine response following acute tibial plateau fracture [J]. J Bone Joint Surg Am.

[36] Terhal P, Venhuizen AJ, Lessel D (2023). AXIN1 bi-allelic variants disrupting the C-terminal DIX domain cause craniometadiaphyseal osteosclerosis with hip dysplasia [J]. Am J Hum Genet.

[37] Alspach E, Lussier DM, Schreiber RD. Interferon gamma and its important roles in promoting and inhibiting spontaneous and therapeutic Cancer immunity [J]. Cold Spring Harb Perspect Biol, 2019, 11(3). 10.1101/cshperspect.a028480 PMC6396335 29661791

[38] Duque G, Huang DC, Dion N (2011). Interferon-gamma plays a role in bone formation in vivo and rescues osteoporosis in ovariectomized mice [J]. J Bone Min Res.

[39] Fortier LA, Barker JU, Strauss EJ (2011). The role of growth factors in cartilage repair [J]. Clin Orthop Relat Res.

[40] Cao Y, Wang H, Zeng W (2018). Whole-tissue 3D imaging reveals intra-adipose sympathetic plasticity regulated by NGF-TrkA signal in cold-induced beiging [J]. Protein Cell.

[41] Wang Y, Spitz MR, Tsou AM (2002). Sulfotransferase (SULT) 1A1 polymorphism as a predisposition factor for lung cancer: a case-control analysis [J]. Lung Cancer.

[42] Sak K, Everaus H (2016). Sulfotransferase 1A1 as a biomarker for susceptibility to carcinogenesis: from Molecular Genetics to the role of Dietary flavonoids [J]. Curr Drug Metab.

[43] Hanson P, Sonesson B (1994). The anatomy of the iliolumbar ligament [J]. Arch Phys Med Rehabil.

[44] Seybold EA, Sweeney CA, Fredrickson BE (1999). Functional outcome of low lumbar burst fractures. A multicenter review of operative and nonoperative treatment of L3-L5 [J]. Spine (Phila Pa 1976).

[45] Khurana B, Sheehan SE, Sodickson AD, Weaver MJ (2014). Pelvic ring fractures: what the orthopedic surgeon wants to know [J]. Radiographics.

[46] Perry K, Mabrouk A, Chauvin BJ. Pelvic Ring Injuries [M]. StatPearls. Treasure Island (FL). 2023. 31335050

