# Supplementary material for: Levels of 91 circulating inflammatory proteins and risk of lumbar spine and pelvic fractures and peripheral ligament injuries: a two-sample mendelian randomization study
Source: J Orthop Surg Res. 2024 Mar 1;19:161. doi: 10.1186/s13018-024-04637-8 (PMC10908089; doi:10.1186/s13018-024-04637-8)
Supplement: Supplementary file 1 — Supplementary Material 1: 91 circulating inflammatory protein number names(Supplementary Table S2) and further details are provided in the Supplementary Information [file 13018_2024_4637_MOESM1_ESM.docx]

| ID | Year | Sex | Population | ncase | ncontral | nsnp | Category |
| --- | --- | --- | --- | --- | --- | --- | --- |
| finn-b-ST19_FRACT_LUMBAR_SPINE_PELVIS(R9) | 2021 | Males and Females | European | 2859 | 212839 | 16380457 | Binary |
| finn-b-ST19_INJURI_ABDOMEN_LOWER_BACK_LUMBAR_SPINE_PELVIS(R9) | 2021 | Males and Females | European | 5953 | 212839 | 16380466 | Binary |

Supplementary Table S1 Details of the included GWASs in this study

Supplemental Figure 1 A-D: Axin-1, BNGF, IFN-gamma and SULT-1A1 of lumbar-pelvic fractures with Leave-one-out test respectively; E-I: BNGF, IFN-gamma, IL-4, MIP-1a and STAM-BP of ligament injuries with Leave-one-out test respectively.

Supplemental Figure 2 A-D: Axin-1, BNGF, IFN-gamma and SULT-1A1 of lumbar-pelvic fractures with forest plots t respectively; E-I: BNGF, IFN-gamma, IL-4, MIP-1a and STAM-BP of ligament injuries with forest plots respectively.

Supplemental Figure 3 A-D: Axin-1, BNGF, IFN-gamma and SULT-1A1 of lumbar-pelvic fractures with funnel plots t respectively; E-I: BNGF, IFN-gamma, IL-4, MIP-1a and STAM-BP of ligament injuries with funnel plots respectively.

Supplementary Table S2 91 circulating inflammatory protein number names

| ID | Reported Trait | Symbol | Efo Traits | Discovery Sample Ancestry | |
| --- | --- | --- | --- | --- | --- |
| GCST90274759 | Adenosine Deaminase levels | ADA | adenosine deaminase measurement | | 14736 European |
| GCST90274760 | Artemin levels | ARTN | artemin measurement | | 11778 European |
| GCST90274761 | Axin-1 levels | AXIN1 | axin-1 measurement | | 11793 European |
| GCST90274762 | beta-nerve growth factor levels | Beta-NGF | beta-nerve growth factor measurement | | 14743 European |
| GCST90274763 | Caspase 8 levels | CASP-8 | caspase-8 measurement | | 14744 European |
| GCST90274765 | C-C motif chemokine 19 levels | CCL19 | C-C motif chemokine 19 measurement | | 14736 European |
| GCST90274766 | C-C motif chemokine 20 levels | CCL20 | C-C motif chemokine 20 measurement | | 14736 European |
| GCST90274767 | C-C motif chemokine 23 levels | CCL23 | C-C motif chemokine 23 measurement | | 14736 European |
| GCST90274768 | C-C motif chemokine 25 levels | CCL25 | C-C motif chemokine 25 measurement | | 14736 European |
| GCST90274769 | C-C motif chemokine 28 levels | CCL28 | C-C motif chemokine 28 measurement | | 14734 European |
| GCST90274770 | C-C motif chemokine 4 levels | CCL4 | C-C motif chemokine 4-like measurement | | 14744 European |
| GCST90274772 | CD40L receptor levels | CD40 | CD40 measurement | | 14736 European |
| GCST90274775 | CUB domain-containing protein 1 levels | CDCP1 | CUB domain-containing protein 1 measurement | | 14734 European |
| GCST90274779 | C-X-C motif chemokine 1 levels | CXCL1 | CXCL1 measurement | | 14736 European |
| GCST90274780 | C-X-C motif chemokine 10 levels | CXCL10 | C-X-C motif chemokine 10 measurement | | 14744 European |
| GCST90274781 | C-X-C motif chemokine 11 levels | CXCL11 | C-X-C motif chemokine 11 measurement | | 14736 European |
| GCST90274782 | C-X-C motif chemokine 5 levels | CXCL5 | C-X-C motif chemokine 5 measurement | | 14736 European |
| GCST90274783 | C-X-C motif chemokine 6 levels | CXCL6 | C-X-C motif chemokine 6 measurement | | 14744 European |
| GCST90274784 | C-X-C motif chemokine 9 levels | CXCL9 | C-X-C motif chemokine 9 measurement | | 14735 European |
| GCST90274777 | Cystatin D levels | CST5 | cystatin-D measurement | | 14736 European |
| GCST90274785 | Delta and Notch-like epidermal growth factor-related receptor levels | DNER | delta and Notch-like epidermal growth factor-related receptor measurement | | 14735 European |
| GCST90274764 | Eotaxin levels | CCL11 | eotaxin measurement | | 14734 European |
| GCST90274758 | Eukaryotic translation initiation factor 4E-binding protein 1 levels | 4EBP1 | level of eukaryotic translation initiation factor 4E-binding protein 1 in blood plasma | | 14736 European |
| GCST90274787 | Fibroblast growth factor 19 levels | FGF-19 | fibroblast growth factor 19 measurement | | 14744 European |
| GCST90274788 | Fibroblast growth factor 21 levels | FGF-21 | fibroblast growth factor 21 measurement | | 14743 European |
| GCST90274789 | Fibroblast growth factor 23 levels | FGF-23 | fibroblast growth factor 23 measurement | | 14735 European |
| GCST90274790 | Fibroblast growth factor 5 levels | FGF-5 | fibroblast growth factor 5 measurement | | 11789 European |
| GCST90274791 | Fms-related tyrosine kinase 3 ligand levels | FIt3L | obsolete_Fms-related tyrosine kinase 3 ligand measurement | | 14734 European |
| GCST90274778 | Fractalkine levels | CX3CL1 | fractalkine measurement | | 14743 European |
| GCST90274792 | Glial cell line-derived neurotrophic factor levels | hGDNF | glial cell line-derived neurotrophic factor measurement | | 14736 European |
| GCST90274793 | Hepatocyte growth factor levels | HGF | hepatocyte growth factor measurement | | 14734 European |
| GCST90274794 | Interferon gamma levels | IFN-gamma | interferon gamma measurement | | 11793 European |
| GCST90274795 | Interleukin-10 levels | IL-1 alpha | interleukin-10 measurement | | 14744 European |
| GCST90274796 | Interleukin-10 receptor subunit alpha levels | IL-10 | interleukin-10 receptor subunit alpha measurement | | 11793 European |
| GCST90274797 | Interleukin-10 receptor subunit beta levels | IL-10RA | interleukin-10 receptor subunit beta measurement | | 14734 European |
| GCST90274798 | Interleukin-12 subunit beta levels | IL10RB | obsolete_interleukin-12 subunit B measurement | | 14735 European |
| GCST90274799 | Interleukin-13 levels | IL-12B | interleukin-13 measurement | | 11792 European |
| GCST90274800 | Interleukin-15 receptor subunit alpha levels | IL-13 | interleukin-15 receptor subunit alpha measurement | | 11792 European |
| GCST90274801 | Interleukin-17A levels | IL-15RA | interleukin-17A measurement | | 11784 European |
| GCST90274802 | Interleukin-17C levels | IL-17A | interleukin-17C measurement | | 11793 European |
| GCST90274803 | Interleukin-18 levels | IL-17C | interleukin 18 measurement | | 14744 European |
| GCST90274804 | interleukin-18 receptor 1 levels | IL-18 | interleukin-18 receptor 1 measurement | | 14743 European |
| GCST90274805 | Interleukin-1-alpha levels | IL-18R1 | obsolete_interleukin-1 alpha measurement | | 11788 European |
| GCST90274806 | Interleukin-2 levels | IL-2 | interleukin-2 measurement | | 11789 European |
| GCST90274811 | Interleukin-2 receptor subunit beta levels | IL-2RB | interleukin-2 receptor subunit beta measurement | | 11792 European |
| GCST90274807 | Interleukin-20 levels | IL-20 | interleukin-20 measurement | | 11784 European |
| GCST90274808 | Interleukin-20 receptor subunit alpha levels | IL-20RA | interleukin-20 receptor subunit alpha measurement | | 11792 European |
| GCST90274809 | Interleukin-22 receptor subunit alpha-1 levels | IL-22RA1 | interleukin-22 receptor subunit alpha-1 measurement | | 11793 European |
| GCST90274810 | Interleukin-24 levels | IL-24 | interleukin-24 measurement | | 11785 European |
| GCST90274812 | Interleukin-33 levels | IL-33 | level of interleukin-33 in blood plasma | | 11793 European |
| GCST90274813 | Interleukin-4 levels | IL-4 | interleukin-4 measurement | | 11793 European |
| GCST90274814 | Interleukin-5 levels | IL-5 | interleukin-5 measurement | | 11792 European |
| GCST90274815 | Interleukin-6 levels | IL-6 | interleukin-6 measurement | | 14743 European |
| GCST90274816 | Interleukin-7 levels | IL-7 | interleukin-7 measurement | | 14736 European |
| GCST90274817 | Interleukin-8 levels | IL-8 | interleukin-8 measurement | | 14744 European |
| GCST90274818 | Latency-associated peptide transforming growth factor beta 1 levels | LAP TGF-beta-1 | transforming growth factor beta-1 measurement | | 14736 European |
| GCST90274819 | Leukemia inhibitory factor levels | LIF | leukemia inhibitory factor measurement | | 11793 European |
| GCST90274820 | Leukemia inhibitory factor receptor levels | LIF-R | leukemia inhibitory factor receptor measurement | | 11784 European |
| GCST90274776 | Macrophage colony-stimulating factor 1 levels | CSF-1 | macrophage colony-stimulating factor 1 measurement | | 14734 European |
| GCST90274825 | Macrophage inflammatory protein 1a levels | MIP-1 alpha | macrophage inflammatory protein 1a measurement | | 14743 European |
| GCST90274826 | Matrix metalloproteinase-1 levels | MMP-1 | matrix metalloproteinase 1 measurement | | 14744 European |
| GCST90274827 | Matrix metalloproteinase-10 levels | MMP-10 | matrix metalloproteinase 10 measurement | | 14744 European |
| GCST90274822 | Monocyte chemoattractant protein 2 levels | CCL2 | monocyte chemotactic protein-2 measurement | | 14736 European |
| GCST90274821 | Monocyte chemoattractant protein-1 levels | CCL8 | CCL2 measurement | | 14733 European |
| GCST90274823 | Monocyte chemoattractant protein-3 levels | CCL7 | monocyte chemotactic protein 3 measurement | | 11783 European |
| GCST90274824 | Monocyte chemoattractant protein-4 levels | CCL13 | monocyte chemotactic protein-4 measurement | | 14736 European |
| GCST90274771 | Natural killer cell receptor 2B4 levels | CD244 | natural killer cell receptor 2B4 measurement | | 14735 European |
| GCST90274829 | Neurotrophin-3 levels | NT-3 | neurotrophin-3 measurement | | 14744 European |
| GCST90274828 | Neurturin levels | NRTN | level of neurturin in blood plasma | | 11791 European |
| GCST90274831 | Oncostatin-M levels | OSM | oncostatin-M measurement | | 14736 European |
| GCST90274830 | Osteoprotegerin levels | OPG | osteoprotegerin measurement | | 14733 European |
| GCST90274832 | Programmed cell death 1 ligand 1 levels | PD-L1 | programmed cell death 1 ligand 1 measurement | | 14736 European |
| GCST90274786 | Protein S100-A12 levels | EN-RAGE | protein S100-A12 measurement | | 14743 European |
| GCST90274835 | Signaling lymphocytic activation molecule levels | SLAMF1 | signaling lymphocytic activation molecule measurement | | 14734 European |
| GCST90274834 | SIR2-like protein 2 levels | SIRT2 | SIR2-like protein 2 measurement | | 14736 European |
| GCST90274837 | STAM binding protein levels | STAMPB | STAM binding protein measurement | | 14736 European |
| GCST90274833 | Stem cell factor levels | SCF | stem Cell Factor measurement | | 14736 European |
| GCST90274836 | Sulfotransferase 1A1 levels | ST1A1 | sulfotrasferase 1A1 measurement | | 11793 European |
| GCST90274773 | T-cell surface glycoprotein CD5 levels | CD5 | t-cell surface glycoprotein CD5 measurement | | 14735 European |
| GCST90274774 | T-cell surface glycoprotein CD6 isoform levels | CD6 | level of T-cell differentiation antigen CD6 in blood plasma | | 14735 European |
| GCST90274845 | Thymic stromal lymphopoietin levels | TSLP | thymic stromal lymphopoietin measurement | | 11793 European |
| GCST90274840 | TNF-beta levels | TNFB | lymphotoxin-alpha measurement | | 11792 European |
| GCST90274844 | TNF-related activation-induced cytokine levels | TRANCE | TNF-related activation-induced cytokine measurement | | 14736 European |
| GCST90274843 | TNF-related apoptosis-inducing ligand levels | TRAIL | TNF-related apoptosis-inducing ligand measurement | | 14735 European |
| GCST90274838 | Transforming growth factor-alpha levels | TGF-alpha | transforming growth factor-alpha measurement | | 14733 European |
| GCST90274839 | Tumor necrosis factor levels | TWEAK | tumor necrosis factor measurement | | 11785 European |
| GCST90274846 | Tumor necrosis factor ligand superfamily member 12 levels | TNF | tumor necrosis factor ligand superfamily member 12 measurement | | 14736 European |
| GCST90274842 | Tumor necrosis factor ligand superfamily member 14 levels | TNFSF14 | tumor necrosis factor ligand superfamily member 14 measurement | | 11793 European |
| GCST90274841 | Tumor necrosis factor receptor superfamily member 9 levels | TNFRSF9 | tumor necrosis factor receptor superfamily member 9 measurement | | 11784 European |
| GCST90274847 | Urokinase-type plasminogen activator levels | uPA | urokinase-type plasminogen activator measurement | | 14734 European |
| GCST90274848 | Vascular endothelial growth factor A levels | VEGF_A | vascular endothelial growth factor A measurement | | 14744 European |
